# Phylogenomics of the archaeal flagellum: rare horizontal gene transfer in a unique motility structure

**DOI:** 10.1186/1471-2148-7-106

**Published:** 2007-07-02

**Authors:** Elie Desmond, Celine Brochier-Armanet, Simonetta Gribaldo

**Affiliations:** 1 Unite Biologie Moléculaire du Gène chez les Extremophiles, Institut Pasteur, 25 rue du Dr. Roux, 75724 Paris Cedex 15, France; 2 Université de Provence Aix-Marseille I, Marseille, France; 3 Laboratoire de chimie bactérienne, Institut de Biologie Structurale et de Microbiologie (CNRS), Marseille, France

## Abstract

**Background:**

As bacteria, motile archaeal species swim by means of rotating flagellum structures driven by a proton gradient force. Interestingly, experimental data have shown that the archaeal flagellum is non-homologous to the bacterial flagellum either in terms of overall structure, components and assembly. The growing number of complete archaeal genomes now permits to investigate the evolution of this unique motility system.

**Results:**

We report here an exhaustive phylogenomic analysis of the components of the archaeal flagellum. In all complete archaeal genomes, the genes coding for flagellum components are co-localized in one or two well-conserved genomic clusters showing two different types of organizations. Despite their small size, these genes harbor a good phylogenetic signal that allows reconstruction of their evolutionary histories. These support a history of mainly vertical inheritance for the components of this unique motility system, and an interesting possible ancient horizontal gene transfer event (HGT) of a whole flagellum-coding gene cluster between Euryarchaeota and Crenarchaeota.

**Conclusion:**

Our study is one of the few exhaustive phylogenomics analyses of a non-informational cell machinery from the third domain of life. We propose an evolutionary scenario for the evolution of the components of the archaeal flagellum. Moreover, we show that the components of the archaeal flagellar system have not been frequently transferred among archaeal species, indicating that gene fixation following HGT can also be rare for genes encoding components of large macromolecular complexes with a structural role.

## Background

Motile archaeal species swim by means of rotating flagellum structures driven by a proton gradient force [1,2], as in bacteria [3]. Interestingly, although they are both responsible for swimming, archaeal and bacterial flagella are not homologous, either in terms of overall structure, components and assembly (for a recent review see [4,5]). The bacterial flagellum is a complex rotary structure made up of as much as 20 proteins and composed of three major parts -the basal body, the hook, and the filament. Rotation is provided by an ATPase exploiting a proton gradient force, and can be switched by specific proteins in response to attractants or repellents in the environment through the chemotaxis system. The filament is a hollow structure about 20 nm in diameter and is composed of a single type of protein called flagellin. Bacterial flagellins are assembled by a complex type III secretion system located in the basal body and are added to the distal tip of the flagellum after passing through the hollow cavity [4].

Much less is known about the archaeal flagellum. It has been extensively studied in terms of components, assembly, and mutation experiments, in Halobacteria and Methanococcales (for recent reviews [4-6]). The archaeal flagellum is a structure thinner than its bacterial counterpart, where at least a filament and a hook are evident [7-9]. The archaeal flagellum has been shown to have a unique symmetry in *Halobacterium salinarium*. In fact, it has 3.3 subunits/turn of a 1.9 nm pitch left-handed helix compared to 5.5 subunits/turn of a 2.6 nm pitch right-handed helix for plain bacterial flagellum filaments [10,11]. The archaeal filament can be made up of different types of homologous flagellin proteins (called FlaA or FlaB). The filament is ~10 nm in diameter and is not hollow, resembling more to bacterial type IV pili in this respect [10]. A few other characteristics of archaeal flagella make them more alike bacterial pili than flagella: as bacterial pilins, archaeal flagellins (i) are made as preproteins with short signal peptides that are processed by a recently identified archaeal-specific signal peptidase (called FlaK) [12-14] that shows weak sequence similarity with the bacterial pili leader peptidase PilD, (ii) are likely added at the base of the filament as in bacterial pili, and (iii) undergo glycosylation as post-translational modification [15] (see [5] for a recent review). Moreover, one component of archaeal flagella (FlaI) is homologous to bacterial PilT, an ATPase involved in bacterial pilin export (a type II/IV secretion system) and pilus retraction during twitching motility [16]. However, none of the remaining archaeal flagellum components are homologous to those of bacterial pili [5]. Moreover, bacterial pili are not rotating structures, and no specific anchoring structures have ever been observed, indicating substantial differences between these two cellular structures.

A number of putative flagellum accessory genes lie close to flagellin genes in archaeal genomes (called *flaC*, *flaD/flaE*, *flaF*, *flaG*, *flaH*, *flaI*, and *flaJ*) [5]. Their putative role in flagellum structure and assembly was tentatively deduced based on their sequences, cellular location, and mutation experiments [4-6].

FlaC, FlaD, FlaH and FlaI are associated with the membrane fraction in *Methanococcus voltae *and may thus be peripheral components of the archaeal flagellum [5,6]. FlaH, FlaI and FlaJ may be important for the assembly of archaeal flagella and possibly form a secretion complex [5,6]. FlaH harbors a domain similar to that found in bacterial RecA-like ATPases, and FlaH mutants are nonmotile and nonflagellated [17]. FlaJ contains many transmembrane domains, while FlaI probably encodes an ATPase that may be important for flagellins export, similarly to the role of its bacterial homologue, the pilin export ATPase PilT, and/or for providing the force for rotation. No experimental data are presently available for FlaG and FlaF, although FlaG may be a component of the anchoring system between the hook and the filament [6]. It may be possible that some of the multiple flagellin proteins have different roles in flagellum substructures other than the filament [6]. Finally, additional components of the archaeal flagellum may be encoded by genes that have not yet been identified.

The uniqueness of the archaeal flagellum in terms of components, structure, and assembly indicates that archaeal and bacterial flagella have distinct origins (i.e. they are analogous systems). Interestingly, homologues of most bacterial chemotaxis genes are found in archaeal genomes [5,18], suggesting that archaeal and bacterial chemotaxis systems are evolutionary related. However, their interaction with the flagellum system in Archaea remains largely unknown (for a recent review see [19]). In this work, we sought to contribute to the research on archaeal flagella and archaeal motility in general performing an accurate phylogenomic study (*sensu *Eisen [20]) of the archaeal motility apparatus in terms of taxonomic distribution of the genes coding for its components, their genomic context, and their phylogeny. This allowed us to sketch a fairly detailed image on the origin and evolution of this macromolecular structure.

## Results

### Taxonomic distribution and genomic context

**Figure 1:**
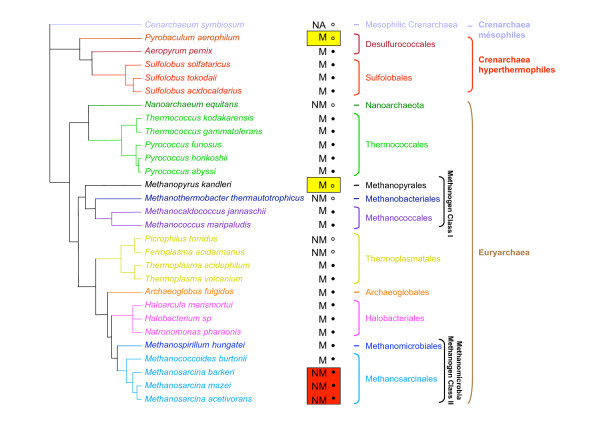
Schematic phylogeny adapted from [29] showing the relationship between the main archaeal phyla for which completely sequenced genomes are available in databases. Motility or non-motility by the mean of a flagellum of each organism according to [21] is indicated by M and NM, respectively (NA is used when no information is available). Black circles and open circles indicate the presence or the absence of flagellum components coding gene in the genomes of the considered organisms, respectively. Red rectangles indicate the presence of flagellum component coding genes in organisms described as non-motile whereas yellow rectangles indicate the absence of flagellum component coding genes in organisms described as motile.

The taxonomic distribution of the genes coding for archaeal flagellum components is congruent with that presented in a recent review[5] and is generally consistent with species descriptions [21]. Gene homologues for all components of the archaeal flagellum are found in the complete genomes of Archaea that are described as motile [5] (indicated by M and ● signs in Figure 1). Conversely, no homologues of genes coding archaeal flagellum proteins are found in the complete genomes of Archaea that are described as non motile [5] (indicated by NM and ○ signs in Figure 1). Although the representative of the *Methanosarcina *genus (i.e. *Methanosarcina mazei*, *Methanosarcina acetivorans *and *Methanosarcina barkeri*) are described as non-motile [21], at least a full complement of homologues of the genes coding for archaeal flagellum components is present in the complete genomes of these species [5,22] (Red rectangles in Figure 1). Conversely, no homologues of the genes coding for archaeal flagellum components are found in the complete genome of *Pyrobaculum aerophilum *[5] (Yellow rectangle in Figure 1). This is surprising since the genus *Pyrobaculum *is described as "motile due to flagellation" in the Bergey's manual and a picture is included of a "platinum shadowed cell showing flagella of *P. aerophilum*" [21]. Similarly, no homologues of the genes coding for archaeal flagellum components are present in the complete genome of *Methanopyrus kandleri *[5] (Yellow rectangle in Figure 1), which is described as motile in the Bergey's manual [21].

**Figure 2:**
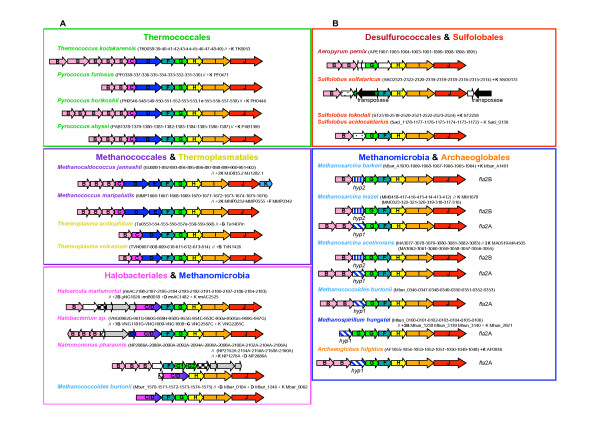
Genomic organization of the genes coding for flagellum components in complete archaeal genomes (*fla *clusters). Numbers within brackets correspond to the locus tags of each gene. A // sign indicates that the following components are elsewhere in the genome. The genes annotated as *hyp*1 and *hyp*2 are probable distant homologues of *flaD/E*. Genes colored in grey are homologues of chemotaxis components, as discussed in the text. Remaining genes where no name is indicated are annotated as hypothetical. **A **Genomic organization of type I clusters (*fla*1). These are found only in Euryarchaea and are characterized by the presence of *flaC *and *flaD/E *and by a conserved gene order *flaA/B*, *flaC*, *flaD*, ***flaF***, ***flaG***, *flaH*, *flaI*, *flaJ*. **B **Genomic organization of the type II clusters (*fla*2). These are found in Crenarchaea and in some Euryarchaea and are characterized by the absence of *flaC *and *flaD/E *and by a conserved gene order *flaA/B*, *flaC*, *flaD*, ***flaG***, ***flaF***, *flaH*, *flaI*, *flaJ*.

In all archaeal genomes harboring flagellum components, the corresponding genes are always organized into one or two very well conserved clusters [5] (*fla *clusters, Figure 2). The only exception is the gene coding for the preflagellin peptidase FlaK, which is located close to the *fla *cluster in *Methanococcus jannaschii *only [5]. *flaK *homologues are nevertheless always present, at least in single copy at different locations in the other archaeal genomes, to the notable exception of *Aeropyrum pernix*, *Thermoplasma acidophilum *and *Thermoplasma volcanium*. We verified that these species do not harbor any homologue of PilD -the bacterial prepilin peptidase- that they may have recruited by horizontal gene transfer, and how they cope with the absence of this enzymatic activity (or which non-homologous enzyme performs the function) remains puzzling.

A careful observation of gene order within each cluster revealed two types of organizations, that we will hereafter call *fla*1 and *fla*2 (Figure 2). *fla*1 clusters are characterized by the presence of *flaC*, plus one or few copies of *flaD *(also annotated as *flaE*), or by a fusion of *flaC *and *flaD *(Figure 2A), whereas *fla*2 clusters lack these genes (Figure 2B). Nevertheless, psi-Blast searches revealed that the genes (*hyp1 *and *hyp2*, Figure 2B) lying between *flaB *and *flaG *in *fla*2 clusters from Methanomicrobia (Methanosarcinales and the Methanomicrobiale *Methanospirillum hungatei*) and Archaeoglobales may be a very distant homologue of *flaD*, as already suggested [5]. A second characteristic differentiates *fla*1 and *fla*2 clusters: they show an inverted order of *flaG *and *flaF*. While the gene order in *fla*1 clusters is *flaB-flaC-flaD-****flaF-flaG****-flaH-flaI-flaJ*, *fla*2 clusters (lacking flaC/D) display the order *flaB-****flaG-flaF****-flaH-flaI-flaJ *(Figure 2). Interestingly, all the archaeal genomes contain only a single type of *fla *clusters (i.e. *fla*1 or *fla*2), except *M. burtonii *that is the only species that harbors both type I and II *fla *clusters. *fla*1 clusters are present only in Euryarchaeota: Thermococcales (*Pyrococcus abyssi, Pyrococcus furiosus, Pyrococcus horikoshii *and *Thermococcus kodakarensis*), Methanococcales (*Methanocaldococcus jannashii *and *Methanococcus maripaludis*), Thermoplasmatales (*Thermoplasma acidophilum *and *Thermoplasma volcanium*), Halobacteriales (*Haloarcula marismortuii, Halobacterium sp. and Natromonas pharaonis*), and Methanomicrobia(*Methanococcoides burtonii*) (Figure 2A); whereas *fla*2 clusters are present in Crenarchaeota: Desulfurococcales (*Aeropyrum pernix*) and Sulfolobales (*Sulfolobus solfataricus Sulfolobus tokodaii Sulfolobus acidocaldarius*) and in some Euryarchaeota: Methanomicrobia (*Methanospirillum hungatei*, *Methanococcoides burtonii*, *Methanosarcina acetivorans*, *Methanosarcina mazei and Methanosarcina barkeri*) and the Archaeoglobale *Archaeoglobus fulgidus *(Figure 2B). In Halobacteriales and Sulfolobales the clusters also include non-flagellum genes (Figure 2A). In particular, a gene coding for a homologue of a bacterial chemotaxis component (MCP domain signal transducer) is present in *Halobacterium sp*., and three genes coding for components of the chemotaxis system are present in *H. marismortui *(CheY, CheA, and CheD) and in *N. pharaonis *(CheY, CheC, and CheD). In this archaeon, the operon is disrupted, the second half lying at ~50 ORFs downstream (Figure 2A). Interestingly,*M. mazei *and *M. acetivorans *each harbor two copies of the *fla*2 cluster (hereafter called *fla*2A and *fla*2B), that differ in the number of flagellin gene copies (two in *fla*2A and one in *fla*2B), and in the presence of two different hypothetical genes (possibly very distant homologues of *flaD*, see above) lying in between the genes coding for FlaB and FlaG (Figure 2B). According to these characteristics, the *A. fulgidus*, the *M. hungatei *and one of the *M. burtonii *gene clusters resemble more to the *fla*2A cluster, while that from *M. barkeri *resembles more to the *fla*2B cluster (Figure 2B). Multiple copies of flagellin genes (*flaB/flaA*) are found in most archaeal genomes, especially in Thermococcales, whereas Thermoplasmatales and Sulfolobales harbor single gene copies (Figure 2), confirming earlier studies on the composition of the flagella from *T. volcanium *and *S. shibatae *[23]. In *M. hungatei*, the flagellin genes lie in another region of the chromosome (two are clustered together and an additional small one is isolated (Figure 2B)), suggesting a disruption of the original cluster. This is also the case of the *fla*1 cluster of *M. burtonii*, which presents no nearby flagellin genes (a single isolated *flaB *gene was possibly part of this cluster before disruption, Figure 2A and see below). In *S. solfataricus *a transposase disrupts the gene coding for FlaG (Figure 2B). However, both the N- and C-ter sequences of FlaG are still very similar to FlaG homologues found in *S. solfataricus *close relatives (e.g. *S. acidocaldarius *and *S. tokodaii*), suggesting that the disruption of *flaG *is recent. Indeed, the sequenced genome of *S. solfataricus *presents indeed a high number of insertion elements that may have recently invaded this strain [24]. Interestingly, cells of this strain appear non-flagellated under the electron microscope (P. Redder, personal communication) even if the disruption of the *flaG *gene does not affect the transcription of the downstream operon genes [25]. This suggests that FlaG (possibly involved in the flagellum anchoring system [6]) is an essential flagellum component.

Finally, additional copies of flagellum components lie in a few instances outside of the clusters (examples are additional *flaB *genes in *M. burtonii*, the two Thermoplasmatales, *H. marismortui *and *N. pharaonis*; an additional *flaG *in *Halobacterium sp*.; an additional *flaD *in *H. marismortui*, *N. pharaonis*, and *M. burtonii*; an additional *flaF *in *M. maripaludis*, and an additional *flaK *in Methanococcales) (Figure 2).

### Phylogenetic analysis

Phylogenetic analyses were performed on six amino acid sequence datasets corresponding to FlaA/B, FlaD/E, FlaG, FlaH, FlaI, and FlaJ. Phylogenetic analysis of FlaC, FlaF and FlaK could not be performed due to a too restricted phylogenetic distribution of FlaC, and the poor sequence conservation of FlaF and FlaK.

#### FlaG, FlaH, FlaI, FlaJ

Among all archaeal flagellum components, FlaH, FlaI and FlaJ are the most conserved at the sequence level and always lie close to each other in all the analyzed genomes (Figure 2) strengthening their likely fundamental role in flagellum assembly and function (see above). FlaI has a number of bacterial homologues belonging to type IV and type II secretion systems, including the typeIV pili component PilT [26]. Moreover, FlaI has additional archaeal homologues that are also probably part of yet to describe secretion machineries [27,28]. In a phylogeny including all these homologues FlaI sequences form a monophyletic group and are most closely related to their archaeal counterparts (not shown). FlaH shares a RecA-like ATPase domain with distant archaeal and bacterial homologues that are not involved in motility structures. Psi-blast searches revealed (i) that FlaJ harbours a few distant archaeal homologues annotated as involved in type II secretion and (ii) weak similarities with the bacterial pilus assembly protein TadC and TadB.

FlaJ shares the domain GSPII F with TadC and TadB. However, this may not be significant, given that the domain was defined on the basis of an alignment that included both archaeal and bacterial sequences. The similarity between the FlaJ sequences and TadB and TadC sequences is very weak (16% and 35% of identity and similarity with TadB sequences, respectively and 14% and 32% of identity and similarity with TadC sequences, respectively) and is mainly the result of the sharing of small hydrophobic amino acids. To our point of view this sequence similarity is too weak to definitively conclude that these sequences are homologues although this has been claimed [27].

**Figure 3:**
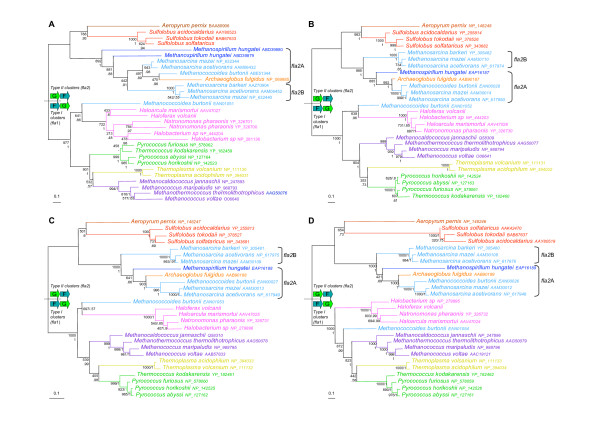
Unrooted maximum likelihood phylogenetic trees of FlaG (**A**), FlaH (**B**), FlaI (**C**) and FlaJ (**D**). Numbers at nodes indicate bootstrap values for 1000 replicates of the original dataset and posterior probabilities, computed by PHYML and MrBayes, respectively. The scale bar represents the average number of substitutions per site. The phylogenies show a clear separation between the type I clusters (characterized by a FlaF FlaG order of genes) and type II clusters (characterized by a FlaG FlaF order of genes).

After removal of ambiguously aligned positions, 104, 193, 392 and 353 amino acids could be kept for phylogenetic analysis of FlaG, FlaH, FlaI, and FlaJ, respectively. The resulting trees are strikingly congruent (Figure 3). Notably, all major archaeal groups except Methanomicrobia (Methanomicrobiales plus Methanosarcinales) are well defined and strongly supported statistically (Bootstrap Values -BV- > 990‰ and/or Posterior Probabilities -PP- = 1), suggesting that no recent horizontal transfer of *flaH*, *flaG*, *flaI *and *flaJ *genes occurred across these groups. The sequences from Methanomicrobia form a well supported cluster (BV > 980‰ and/or Posterior Probabilities -PP- = 1) that also includes sequences from *A. fulgidus*. It is not possible for the time being to decide whether this is due to a HGT or a hidden paralogy. Importantly, the corresponding trees are also strongly consistent with gene cluster organization. In fact, homologues from *fla*1 and *fla*2 clusters (characterized by a *flaF*-*flaG *and by a *flaG*-*flaF *gene order, respectively) form two distinct strongly groups (BV > 975‰ and PP = 1, Figure 3). In particular, in all four trees, the homologues from the *fla*2 clusters of *M. hungatei*, the four Methanosarcinales and *A. fulgidus *appear close to Crenarchaeota (Figure 3). This is in contrast to their expected position as sister-group of Halobacteriales within Euryarchaeota (Figure 1, [29,30]). Interestingly, such expected position is shown by the *M. burtonii *sequences belonging to its *fla*1 cluster (Figure 2A). This suggests that the *fla*1 and *fla*2 clusters from *M. burtonii *have different origins (see below). Independent species-specific duplications of *flaG *appear to have occurred in *Halobacterium sp*., *N. pharaonis*, and *M. hungatei *(tandem gene duplications in these last two species). Moreover, in the FlaH, FlaI and FlaJ phylogenies, the sequences from *M. burtonii*, *M. hungatei, M. barkeri *and *A. fulgidus fla*2 clusters group with the sequences belonging to the *fla*2B cluster from *M. mazei *and *M. acetivorans *(Figures 3B, 3C and 3D, respectively), supporting a close relationship of these clusters, as suggested by their gene organization (Figure 2B).

#### FlaD/E

**Figure 4:**
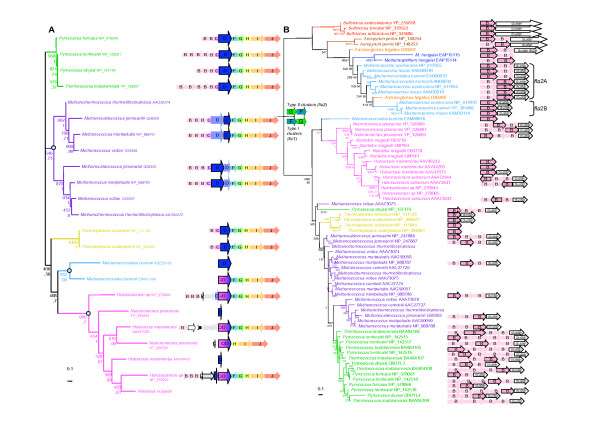
**A**. Unrooted maximum likelihood phylogenetic trees of FlaD/E. Numbers at nodes indicate bootstrap values for 1000 replicates of the original dataset and posterior probabilities, computed by PHYML and MrBayes, respectively. The scale bar represents the average number of substitutions per site. The light blue circles indicate duplication events. **B**. Unrooted maximum likelihood phylogenetic trees of FlaA/B. Numbers at nodes indicate bootstrap values for 1000 replicates of the original dataset and posterior probabilities, computed by PHYML and MrBayes, respectively. The scale bar represents the average number of substitutions per site. Detailed cluster organizations are not shown. White arrows are used to schematize the clusters. The phylogenies show a clear separation between the type I clusters (characterized by a FlaF FlaG order of genes) and type II clusters (characterized by a FlaG FlaF order of genes).

As discussed above, homologues of FlaD/E genes are missing in all *fla*2 clusters from Crenarchaea. In *fla*2 clusters from Methanomicrobia and *A. fulgidus *the two hypothetical proteins *hyp1 *and *hyp2 *could be distantly related to FlaD/E (Figure 2B). However they are too distant to be included in any phylogenetic analysis. The small number of unambiguously aligned positions (77 amino acids positions) that could be kept for phylogenetic analysis gives a poor resolution of the relationships between major euryarchaeal groups (Figure 4A). However, sequences from phyla form monophyletic groups generally well supported (BV = 996‰, PP = 0.93, BV = 1000‰ and BV = 958‰ for Halobacteriales, Methanosarcinales, Thermoplasmatales and Thermococcales, respectively, Figure 4A). This indicates that, similarly to FlaG, FlaI, FlaJ, and FlaH, no recent HGT involving the *flaD/E *gene occurred among these groups. Interestingly, a tandem duplication event appears to have occurred in an ancestor of Methanococcales, with the two copies having been conserved within the cluster. Halobacteriales also harbor two copies of *flaD*. One of the two copies is fused with *flaC *and always resides within the *fla *cluster, whereas the second copy resides within the fla cluster only in *Halobacterium sp*. This suggests that the fusion of *flaC *and *flaD *genes occurred before the divergence of the three Halobacteriales, but after the duplication event and that one of the two copies was displaced after the duplication event in the ancestor of *H. marismortui *and *N. pharaonis*. A similar duplication of FlaD followed by a fusion of one of the two copies of FlaD and FlaC also appears to have occurred in *M. burtonii*. As in most Halobacteriales one of the two copies resides outside the *fla *cluster (Figure 2A) suggesting its displacement after the duplication event. The poor resolution of the relationships between groups (due to the small number of positions kept for the phylogenetic analysis) does not permit to determine if a single fusion event of FlaC and FlaD occurred in ancestor of Halobacteriales and Methanomicrobia or if this event occurred two times independently in both lineages.

#### FlaB

Given the use of only 72 unambiguously aligned positions for analysis, the FlaB tree presents a number of poorly resolved nodes (Figure 4B). Nevertheless, the monophyly of a number of groups is recovered and supported by BV > 850‰, except for Thermococcales, Methanococcales and Methanomicrobia. As for FlaG, FlaI, FlaJ, and FlaH, the FlaB tree is again strongly consistent with gene cluster organization (Figure 4B). In particular, the FlaB from the *fla*2 clusters of *M. hungatei*, the four Methanosarcinales and *A. fulgidus *appear close to Crenarchaeota, while the isolated FlaB from *M. burtonii *appear close to Halobacteria, and thus likely functions with the flagellum components of *fla*1 cluster (Figure 4B). Interestingly, the multiple copies of flagellins group in a group-specific manner (Figure 4B), suggesting that flagellin gene family expansion occurred mainly by gene duplication and multiple times independently in different species, and not by HGT.

## Discussion and conclusion

**Figure 5:**
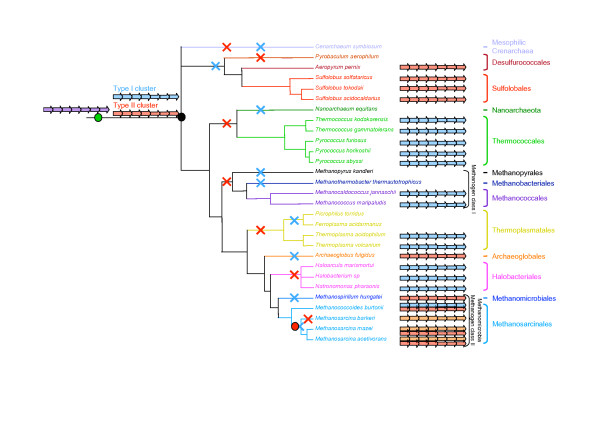
An evolutionary scenario for the origin and evolution of the archaeal flagellum. Blue, red, orange and purple clusters represent *fla*1 cluster, *fla*2A cluster, *fla*2B cluster and their ancestor, respectively. The black circle represents the last common ancestor of Archaea. The green circle represents the duplication event that led to the *fla*1 and *fla*2 clusters. This duplication event occurred before the last common ancestor of Archaea, which thus harbored the two types of clusters. The red circle represents the recent duplication event of the *fla*2 cluster in ancestor of *Methanosarcina*. The blue and red crosses represent the loss of the *fla*1 and *fla*2A clusters, respectively.

**Figure 6:**
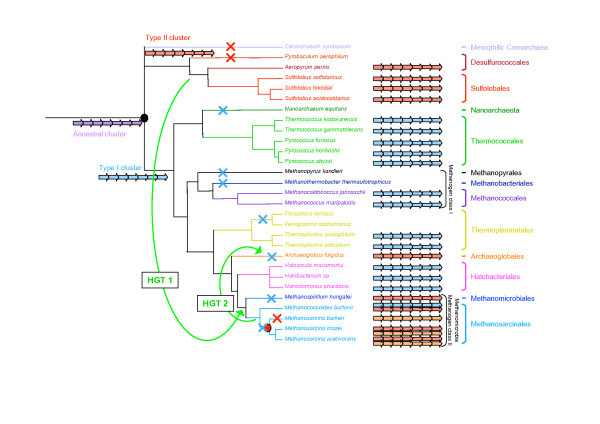
An evolutionary scenario for the origin and the evolution of the archaeal flagellum. Blue, red, orange and purple clusters represent *fla*1 cluster, *fla*2A cluster, *fla*2B cluster and their ancestor, respectively. The black circle represents the last common ancestor of Archaea. The red circle represents the recent duplication event of the *fla*2 cluster in ancestor of *Methanosarcina*. The blue and red crosses represent the loss of the *fla*1 and *fla*2A clusters, respectively. The green arrows represent horizontal gene transfers.

**Figure 7:**
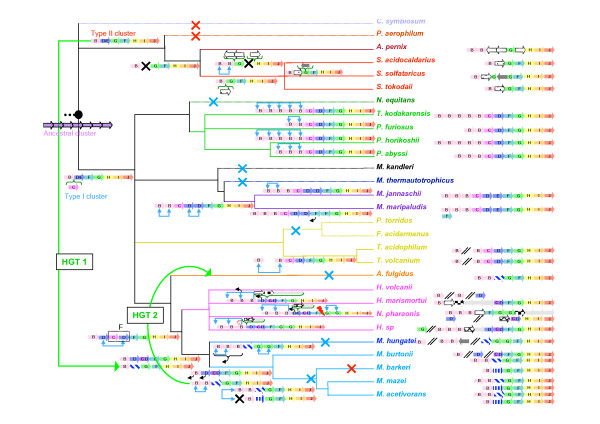
A detailed evolutionary scenario for the origin and the evolution of the archaeal flagellum based on figure 6. The purple cluster represents the ancestor of *fla*1 and *fla*2 clusters. The black circle represents the last common ancestor of Archaea. Blue arrows represent the recent duplication events. Black arrows indicate gene movements to different locations from their original positions in the cluster. Dark green { symbols indicate gene insertions within the clusters. The blue, red and black crosses represent the loss of the *fla*1 cluster, *fla*2A cluster, or of single genes, respectively. A red arrow indicates gene inversion. F indicates the fusion of FlaC and FlaD. The green arrows represent horizontal gene transfers.

The archaeal flagellum is a complex cellular structure composed of multiple subunits and anchored to the membrane. The striking conservation of the genomic context of the genes coding for these subunits indicates a likely highly coordinated expression and assembly mechanisms. Coupled to the high sequence conservation of the different subunits across archaeal species inhabiting very different habitats, this underlines the importance for structural maintenance of the archaeal flagellum. However, we highlighted an important dimorphism of the genetic context organization, with two types of gene clusters harboring differences in both gene content and gene order (Figure 2). In fact, most Euryarchaea exhibit the *fla*1 cluster, whereas all the Crenarchaea and some Euryarchaea have the *fla*2 cluster (e.g. Methanomicrobia and *A. fulgidus*). The difference between the two clusters is strongly supported by phylogenetic analysis, and indicates that Methanomicrobia and *A. fulgidus *flagellum components encoded by *fla*2 gene clusters are more closely related to their crenarchaeal homologues than to the homologues encoded by the *fla*1 gene cluster of their close relatives (i.e. *M. burtonii*, Thermoplasmatales and Halobacteriales, Figures 1, 3 and 4). Two different evolutionary scenarios can be proposed to explain our results. In the first scenario (Figure 5), the last ancestor of Archaea was flagellated and had the two types of clusters (i.e. both *fla*1 and *fla*2, blue and red clusters, respectively, Figure 5), resulting from the duplication of an ancestral cluster (purple cluster, Figure 5), and these were secondarily and differently lost during archaeal lineages evolution (seven losses of the *fla*1 cluster and nine losses of the *fla*2 cluster). Importantly, some of these losses would have also occurred recently in the Euryarchaea (for example the loss of the *fla*1 cluster in the Methanosarcinale lineage would have occurred after the divergence of *M. burtonii*, that would have kept the two types of clusters). Finally, a duplication event of the whole *fla*2 cluster occurred in an ancestor of *Methanosarcina *(red circle, Figure 5) leading to the *fla*2B cluster (orange cluster, Figure 5). This first scenario involves 16 losses, and implies that the ancestor of Archaea and the ancestor of each euryarchaeal group had two types of flagella. Moreover, it does not explain the position of *A. fulgidus *sequences within the Methanomicrobia group and not as sister of this group, as generally indicated by molecular phylogeny of multiple markers (Figure 1 and [30]). A second scenario involves less losses (three losses of the *fla*2 cluster and seven losses of the *fla*1 cluster) (Figure 6 and Figure 7 for a more detailed scenario). Here, the ancestor of Archaea was also flagellated, but had only a single type of *fla *cluster (either *fla*1, *fla*2, or else, purple cluster in Figure 6 and Figure 7). After the divergence of Crenarchaea and Euryarchaea (black circle, Figure 6 and Figure 7), evolution led to the *fla*1 and *fla*2 clusters. A single HGT of the *fla*2 cluster would have then occurred from some ancestors of Sulfolobales and Desulfurococcales to an ancestor of Methanomicrobia (Figure 6 Figure 7, HGT 1), and was followed by a HGT from some ancestors of Methanosarcinales to *A. fulgidus *(Figure 6 Figure 7, HGT 2). *Methanosarcina*, *M. hungatei *and *A. fulgidus *would thus have lost their original *fla*1 gene cluster before or after their replacement by a *fla*2 gene cluster of crenarchaeal origin. We favor a HGT from Crenarchaea to Methanomicrobia rather than the opposite, since in this case we would expect to find the *M. burtonii fla*1 genes more closely related to their paralogues belonging to the *fla*2 cluster than to their *fla*1 orthologues from Halobacteriales.

Both scenarios imply that the archaeal flagellum would have appeared prior to the last archaeal ancestor

Apart these two likely cases of HGT of the entire *fla*2 gene cluster, we found no clear evidence for recent transfers of the genes coding for flagellum components among archaeal species. Indeed, the poor resolution of inter-phyla relationships in some trees is more likely due to lack of phylogenetic signal rather than horizontal gene transfer. One explanation for the rarity of HGT is that it is possible the result of the high level of integration of flagellum components within the macromolecular structure. Importantly, this contradicts the generally assumed notion that HGT of "informational" genes (i.e. those coding for proteins involved in the expression and the transmission of genetic information) are less frequent than those involving the remaining ones ("operational") genes. Nevertheless, the acquisition of a whole flagellum component coding gene cluster from distant donors seems possible. Interestingly, even if two clusters coexist within a genome (i.e. in *M. burtonii*) neither gene recombination is observed between homologous genes belonging to the two clusters, nor losses, suggesting that the components of a cluster may interact preferentially due to coevolution, although they form similar cellular structures. The presence in *M. burtonii *of two types of *fla *clusters (possibly one native and one acquired by HGT from crenarchaeota) represents an interesting experimental model to study. It would be in fact particular interesting to know the difference between the components encoded by the two *fla *clusters in terms of expression and assembly, and how two different flagellum systems coexist in this archaeon.

Finally, it has been suggested that archaea are modified Actinobacteria and that archaeal flagella are derived from bacterial flagella following an adaptation to acidic environments by the recruitment of an already acid-stable glycoprotein from the pilus machinery that would have replaced the original flagellin while leaving intact the basal rotary motor [31]. We find this hypothesis unlikely for the fact that archaeal flagella do not resemble to bacterial flagella in major structure, assembly, and components, and not only concerning flagellin. Indeed, no even distant homologues to any component, including the basal rotary motor, of bacterial flagella can be recovered in archaeal genomes, including the related type III secretion system components [28]. Moreover, flagella of acidophilic bacteria (such as *Thiobacillus*) show a typical bacterial structure (e.g. a diameter of approximately 20 nm), so they adapted to acidic conditions without radically modifying their motility structures and components [28]. Indeed, the uncomplete genome of the extreme acidophilic bacterium (optimal pH<3) *Acidobacterium capsulatum *contains a clear homologue of bacterial flagellin, indicating that adaptation to acidic environments was possible without its replacement.

The lack of congruence between the description of archaeal species as motile or non-motile and the taxonomic distribution of homologues of flagellum component coding genes[5] is particularly striking and underlines the need to explore more in depth the motility systems in Archaea. The presence of two gene clusters in non motile Methanosarcinales is particularly puzzling. Either these species can be flagellated under particular conditions that have not yet been tested, or the flagellum component homologues are involved in other functions than motility (for example, they could be required for cell-cell adhesion to form cell aggregates, a peculiarity of this archaeal family). It will be extremely interesting to study the expression and localization of the flagellum components in Methanosarcinales, in the light of the fact that the flagellum genes of Methanosarcinales may have been recruited from the distantly related Crenarchaeota, since this event may have been an important step in the evolution of this archaeal lineage. Moreover, virtually nothing is known about other types of motility than swimming in archaea [28], while in bacteria these are starting being investigated [4]. The fact that no homologues of flagellum components are encoded in the genomes of at least two archaeal species that are described as motile (*M. kandleri *and *P. aerophilum*) is also puzzling. Although it is possible that the strains used for genome sequencing have lost the flagellum operon following lab cultivation (see for example [32], it would surely be interesting to test motility in these archaeal species. Alternatively, this observation could also suggest that other types of motility may occur in archaea and are made possible by still unknown molecular structures. It would also be very useful to investigate the relationship between the flagellum and the secretion systems in Archaea. Indeed, archaeal genomes harbor only a few homologues of bacterial TypeII/IV secretion systems [28], and it is not known whether they form pili, despite rare observations [33-35] and evidence for conjugation [36-38].

To sum up, two radically different options for motility were adopted at the divergence of the two prokaryotic domains, and much still remain to be uncovered on archaeal motility systems.

## Methods

### Data set construction

The list of archaeal flagellum components was retrieved from the Kyoto Encyclopedia of Genes and Genomes [39]. Homologous sequences of each archaeal flagellum component were retrieved from the *nr *database at the National Center for Biotechnology Information [40] using the BlastP program with different seeds [41] and the ALIBABA program (P. Lopez personal communication). For each dataset, additional searches using psi-Blast were performed to search for divergent homologues (especially from bacteria) [41]. tBlastN searches on the unfinished genomes of the Halobacteriale *Haloferax volcanii *were performed at TIGR [42]. Multiple alignments were done with ClustalW [43] and MUSCLE [44]. For each dataset, the quality of the alignments obtained with CLUSTALW and MUSCLE, was evaluated with T-COFFEE (CLUSTALW and MUSCLE provided alignments of comparable quality, not shown) [45]. All the alignments were edited and manually improved using the ED program from the MUST package [46]. Regions where the homology between sites was doubtful were manually removed from the datasets for phylogenetic analyses.

### Phylogenetic analysis

Maximum Likelihood (ML) phylogenetic trees were computed with Phyml [47,48] and the JJT model of amino acid substitution (Jones, Taylor and Thornton [49]. A gamma correction with 4 discrete classes of sites was used to take into account the heterogeneity of evolutionary rates across sites. The alpha parameter and the proportion of invariable sites were estimated for each dataset. The robustness of each branch was estimated by non-parametric bootstrap analysis (1000 replicates) using PHYML. In addition, bayesian analyses were performed using MrBayes [50] with a mixed model of amino acid substitution. As for ML tree reconstruction, a gamma correction with 4 discrete classes of sites was used and the alpha parameter and the proportion of invariable sites were estimated. MrBayes was run with four chains for 1 million generations and trees were sampled every 100 generations. To construct the consensus tree, the first 1500 trees were discarded as "burnin".

### Genomic context analysis

The genomic localization of each archaeal flagellum component was manually investigated in all archaeal complete genomes available at the NCBI.

## Authors' contributions

SG conceived the study and supervised the analyses. ED carried out the analyses. CB and SG refined and completed the analyses and wrote the manuscript. All authors read and approved the final manuscript.
